# So Near and Yet So Far: Harmonic Radar Reveals Reduced Homing Ability of *Nosema* Infected Honeybees

**DOI:** 10.1371/journal.pone.0103989

**Published:** 2014-08-06

**Authors:** Stephan Wolf, Dino P. McMahon, Ka S. Lim, Christopher D. Pull, Suzanne J. Clark, Robert J. Paxton, Juliet L. Osborne

**Affiliations:** 1 Department of Agro-Ecology, Rothamsted Research, Harpenden, Hertfordshire, United Kingdom; 2 School of Biological and Chemical Sciences, Queen Mary University of London, London, United Kingdom; 3 School of Biological Sciences, Queen’s University Belfast, Belfast, Northern Ireland, United Kingdom; 4 Institute for Biology, Martin-Luther-Universität Halle-Wittenberg, Halle/Saale, Germany; 5 IST Austria (Institute of Science and Technology Austria), Klosterneuburg, Austria; 6 Department of Computational and Systems Biology, Rothamsted Research, Harpenden, Hertfordshire, United Kingdom; 7 Environment and Sustainability Institute, University of Exeter, Falmouth, Cornwall, United Kingdom; University of Guelph, Canada

## Abstract

Pathogens may gain a fitness advantage through manipulation of the behaviour of their hosts. Likewise, host behavioural changes can be a defence mechanism, counteracting the impact of pathogens on host fitness. We apply harmonic radar technology to characterize the impact of an emerging pathogen - *Nosema ceranae* (Microsporidia) - on honeybee (*Apis mellifera*) flight and orientation performance in the field. Honeybees are the most important commercial pollinators. Emerging diseases have been proposed to play a prominent role in colony decline, partly through sub-lethal behavioural manipulation of their hosts. We found that homing success was significantly reduced in diseased (65.8%) versus healthy foragers (92.5%). Although lost bees had significantly reduced continuous flight times and prolonged resting times, other flight characteristics and navigational abilities showed no significant difference between infected and non-infected bees. Our results suggest that infected bees express normal flight characteristics but are constrained in their homing ability, potentially compromising the colony by reducing its resource inputs, but also counteracting the intra-colony spread of infection. We provide the first high-resolution analysis of sub-lethal effects of an emerging disease on insect flight behaviour. The potential causes and the implications for both host and parasite are discussed.

## Introduction

Behavioural changes in the host in response to infection, whether as a side-effect of infection or an active manipulation by the pathogen or a behavioural response of the host, can have profound direct and indirect effects on both parasite and host [1]. Such behavioural effects may actively facilitate the transmission of the pathogen, such as by active exposure to susceptible hosts or navigational deficits leading to higher encounter rates with new prospective hosts (e.g. ‘drifting’ of honeybees between colonies) (reviewed in [1]). Likewise, pathogen-induced behaviours may be a defence mechanism of the host limiting the spread of the pathogen (e.g. [2]). Thus behavioural changes may be important in shaping host-parasite interactions beyond its immediate effects on both the host and the parasite.

Social insects typically live at high density in a relatively homogenous environment in the colony and are in many cases genetically closely related; these provide ideal conditions for many pathogens (e.g. [3]). In the honeybee alone, 71 parasite species have been identified [1]. Therefore, social insects are a prime model to study the behavioural effects of pathogens and their consequences, additionally emphasised by the enormous economic and ecological roles some of these species have as pests (e.g. ant species, termites) and as pollinators (e.g. bees).

Honeybees (*Apis mellifera* L. 1758) are the most intensively managed insect pollinators [4]–[6] and are important for the pollination of many crops [7]. As an example, over one million hives alone are used per year to pollinate almonds in California and sustain the Californian almond industry, which is valued at around $2 billion per year [8]. Although the pollination service provided by honeybees may be compensated by wild pollinators in some agricultural systems [6], [9]–[11], the absence of honeybees may lead to substantial reductions in yield of up to 50% or more [4], [12] and would cause dramatic agricultural and economic impacts.

The importance of the honeybee, however, is harshly contrasted by continued high death rates of colonies, especially in North America and Europe (e.g. [8], [13]–[16]), which have become the focus of intensive scientific scrutiny (e.g. [16], [17]). As yet, no single explanatory factor for the observed high losses of honeybee colonies has been identified, and the current consensus is that colony failure is a complex problem, with a multitude of interacting factors being responsible [18]. Emergent and exotic pests and diseases such as *Varroa destructor* and *Nosema ceranae* are thought to be one such component in driving colony losses (e.g. [8], [16], [19]).

Effective mitigation strategies crucially depend on an understanding of the effects of pathogens on the performance of individual bees and colonies. However, only sometimes do these effects manifest themselves with obvious clinical symptoms, as in American foulbrood (*Paenibacillus larvae larvae*; [20]) that kills larvae. More often, pathogens affect their hosts in more subtle and cryptic but nonetheless pernicious ways. Such sub-lethal effects are notoriously difficult to study and can often only be quantified in controlled laboratory experiments. This is particularly true for effects on the “power house” of a colony, its foraging worker force. Foragers spend considerable time outside of the hive and disperse widely in the landscape, limiting time and opportunities for the collection of high quality data.

*Nosema ceranae* is a microsporidian gut parasite first found in the Eastern Honeybee (*Apis cerana*; [21]) that has successfully invaded colonies of *A. mellifera* as an alternative host after being anthropogenically dispersed across the world (e.g. [22]–[24]). In some regions it is suggested to be a major cause of *A. mellifera* colony losses [22], [25], [26]. Though *N. ceranae* may arguably be more virulent than its European counterpart *N. apis* [25], [27]–[29] (but see [30]) and even fatal for colonies when at high prevalence, its effects on both individual and colony are generally more subtle, with no obvious symptoms before death [29].

This pathogen has, however, been demonstrated to have discernible effects on individual honeybee behaviour [31]–[33]. Investigating the physiology of *Nosema*-infected bees, Mayack & Naug (2009) [32] showed an increase in energy consumption, lowering response thresholds to different concentrations of sucrose (a proxy measure for hunger) and significantly shortening honeybee lifespan when energy limited. Energetic stress also affected intra-colonial social behaviour; infected bees shared proportionally less food with their nest-mates as compared to uninfected bees [33]. At the colony level, less interactive bees may represent a host defensive response compromising pathogen transmission. Dussaubat et al. (2013) [34] also found increased flight activity in *Nosema*-infected bees, arguing that this may help to counteract within-colony transmission. Kralj & Fuchs (2009) [31] reported significantly increased losses and prolonged homing times for infected foragers on forced homing flights as short as 30 m, again potentially limiting within-colony pathogen transmission; they also showed that infected bees approach decoy entrances significantly more often than uninfected foragers, suggesting an impairment of the bees’ orientation abilities [31], potentially enhancing transmission of *Nosema* into other colonies in the vicinity.

In this study, we explore the impact of pathogen infection on homing performance and navigation of honeybee foragers, which are vitally important to colony welfare and growth [35]. We conduct a homing flight experiment, which is a simple yet effective method for investigating the abilities of bees to find their colony using memorized landscape features (reviewed in [36]; [37]–[39]). Using harmonic radar, we were able to go beyond just the *result* of the flight (namely homing success and homing time) and study the entirety of a honeybee’s flight, including its navigational performance. Radar tracking has been successfully employed to study recruitment precision [40], orientation flights [41]–[45], search patterns [46]–[48], spatial scale [49], optimization of bee flights [50], bee homing performance following pesticide exposure [51] and flight patterns of butterflies [52], [53]. However, this technology has so far not been utilized to specifically study the flight performance of bees in the context of their disease burden. Employing radar tracking technology, here we provide a novel insight into the sub-lethal effects of *N. ceranae* on the flight patterns of free flying honeybee foragers.

We expected to find that *Nosema*-induced energetic stress, previously demonstrated in the laboratory [32], also affect free-flying bees, resulting in slower flights and/or a more pronounced flight discontinuity, ultimately limiting the bee’s ability to return to the hive and thus limiting within-colony spore transmission. We also expected *Nosema*-infected bees to show signs of disorientation [31], such as taking flights in the wrong direction, intensified searching behaviour or reduced ability to use natural landmarks for orientation, compared to healthy bees, potentially facilitating transmission of the pathogen to new host colonies.

## Materials and Methods

### Bees

All experimental bees originated from three quarantined (see file S1 for details) donor colonies (DCs) from the Isle of Colonsay, UK (56° 3′ 56″ N, 6° 12′ 24″ W), which is currently devoid of *Varroa destructor* so that potentially confounding effects of this mite or its associated viruses were minimized. Regular inspections for *Varroa* and *Nosema* ensured the continual absence of these parasites in the quarantined DCs throughout the season.

Experimental bees were obtained from mature capped brood frames taken from DCs and transferred into an incubator (34.5°C) until the bees emerged. Two to three-day old worker bees were collected from these brood frames, randomly allocated to one of three treatment groups and marked individually with Opalith numbered labels (Bienen-Voigt and Warnholz GmbH and Co. KG, Germany). Each bee received a 10µl treatment-specific inoculum (see below) and was transferred to one of three holding cages in an incubator. After a minimum of two hours, all three groups of bees were introduced into a medium sized *A. mellifera* host colony (HC), where they could develop into foragers under natural conditions and *Nosema* can establish in the host. The HC was equipped with a Perspex entrance tunnel with several shutters, allowing for better visual detection, separation and access to foraging experimental bees. The HC was regularly monitored for the presence of the queen, brood and stores. With typical foraging activity and expected colony build-up, this colony showed no sign of atypical behaviour that could have interfered with the experiment during the course of observations.

### Treatments

*Nosema ceranae* spores were extracted from the guts of 5–7 infected workers. After purification and quantification (see file S1 for detailed description), the spore solution was mixed with a 40% sucrose solution (w/w), forming the treatment inoculum (hereafter Nc). Then 100,000 *N. ceranae* spores (ca. 10 µl solution) were fed using a pipette to a bee harnessed in a cage. The spore samples were repeatedly checked for *Nosema* species identity using a species-specific PCR [54].

We employed two different control treatments. First we used *Nosema*-free gut-extracts (GE), potentially containing traces of the natural gut flora of bees. Purification and preparation of the parasite-free gut extract inoculum (hereafter C_GE_) was carried out in parallel to the Nc-inoculum, following an identical protocol. All C_GE_-samples were carefully examined microscopically to ensure the absence of *Nosema* spores before being added to a sucrose solution. The second control group received 10µl of pure 40% sucrose solution (C_S_).

Post-hoc *Nosema* screening of all tracked and retrieved bees (see below) was carried out microscopically. Only screening of the spore loads from the dissected mid-gut was carried out as defecation potentially reduces spore loads in the hind-gut.

### Harmonic Radar

The tracking of a bee’s flight relies on a 16 mm long dipole aerial with a Schottky diode, forming a transponder that is vertically attached to the thorax of the bee [55]. At ca. 12–15 mg, the weight of the transponder is considerably lighter than a typical nectar or pollen load carried by a bee [41].

The transponder is excited by microwaves emitted from a stationary, horizontally scanning radar system (9.41 GHz-transmitter, 3 kHz pulse repetitive frequency (PRF)) and returns a microwave of a harmonic frequency of the original wave for which the experimental arena is specifically scanned. With the radar system turning at 20 rpm, this provides a positional record of a bee flying within a range of 900 m every 3 seconds [55], [56]. The transponder signals are not uniquely identified and only one individual can be tracked at a time. Radar tracking relies on a clear line-of-sight between the radar and the tracked object. Obscuring landscape features like high vegetation, buildings or high terrain may prevent the continuous recording of positional information [49].

### Experimental Design

**Figure 1:**
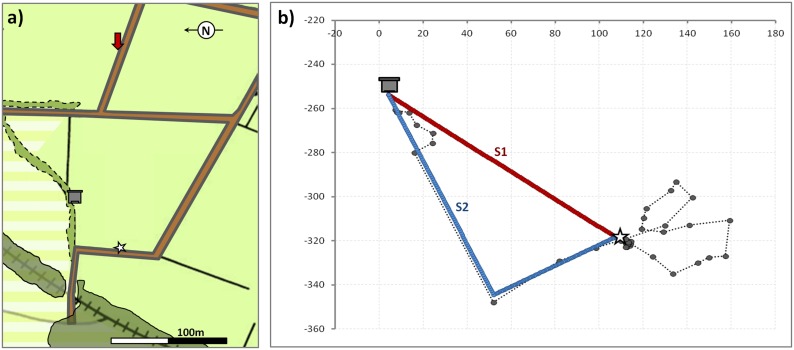
Study area. a) Schematic map of the study area indicating the positions of the experimental colony (hive-symbol), the release site (star) and the radar (red arrow) in the agricultural landscape (non-flowering crop/harvested fields: light green; field margins: solid black line; field tracks: brown; hedges: green, dashed black outline; woodland: dark green, solid black outline). Areas with impaired radar tracking are shown in horizontal stripes. b) Graphical representation of a homing flight track (circles) and the two hypothetical flight scenarios in a radar-centred x-y-coordinate system (unit = metres): S1) straight homing flight from the release site (star) to the colony (red) and S2) flight along available field margin connecting the release site and the hive (blue), both represented by the coordinates of 100 points evenly distributed along either path. The distance of the realized homing flight to either scenario was used as a proxy measure of the utilization of landscape features by bees of the different treatments to orientate towards the colony.

The experiment was undertaken in favourable weather conditions (ambient temperature on average >15°C, no rain, no or light wind) between 15^th^ July and 27^th^ September 2011 across a flat and harvested wheat field at Rothamsted Farm, UK (51° 48′47.44″ N, 0° 22′ 45.74″ W), providing ideal conditions for unobstructed radar tracking over several hundred metres. Positions of the host colony (HC), the radar (R) and the release site (RS) are given in Fig. 1a. The host colony was located at a field margin where it was sheltered by a hedge but sufficiently prominent to allow bee tracking even close to the entrance of the hive. The release site (RS) was located 120 m across a harvested wheat field from the hive. Though within the familiar landscape, the exact position of the RS did not provide any visual cue that the bees could have learned as a conspicuous landmark prior to tracking.

Marked returning foragers (treated and control) were collected from the entrance tunnel using a small cage, and we ensured an approximately even contribution of groups throughout the day. A caged bee was placed in a dark box and transferred to the release site, where the bee was equipped with the transponder and released immediately. By using returning foragers, bees were naturally motivated to return to the colony and unload collected nectar and pollen, ensuring tracked bees performed a flight towards the colony rather than embarking on a foraging flight. This approach also allowed us to account for the behavioural age of the bees (first forging flights typically occur 12 days after emergence, [57]), reducing the chances of tracking inexperienced bees. Active foraging typically commences only after a period of close range orientation during which the landscape features around the hive are learned [41]. Therefore, incoming foragers can be assumed to have a well established memory of the colony position in the surrounding landscape.

**Figure 2:**
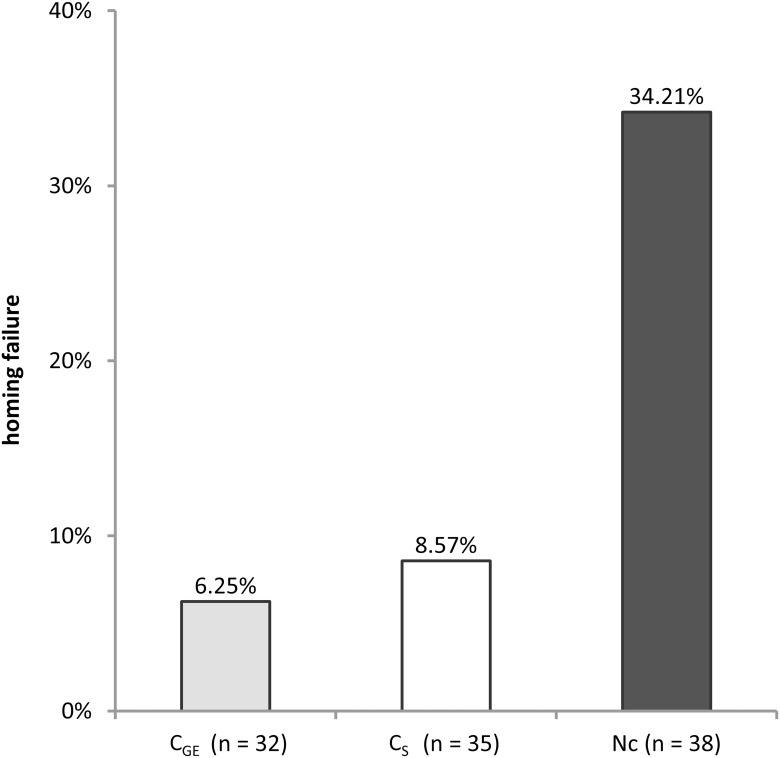
Homing performance. Percentage of non-returning bees during homing flights per treatment group (sample sizes: Nc: 38; C_GE_: 32; C_S_: 35). A significantly higher proportion (*X^2^* = 12.3, *d.f.*  = 2, *p* = 0.002) of bees inoculated with *Nosema ceranae* failed to return to the colony as compared to the control bees.

The time required to catch, displace and prepare the bee (hereafter referred to as ‘handling time’) was recorded using a stopwatch. Despite the coordinates of the release site remaining constant throughout the experiment, we ensured a bee’s start position was recorded by the radar prior to release to radar-record the actual bee position, to ensure the functionality of the transponder and to provide an accurate start point for each track. During homing flights, the entrance of the colony was closely monitored. Bees that successfully returned to the HC (Fig. 2) were caught before they entered the hive and the transponder was removed. All tracked and successfully retrieved bees were kept in individual 50 ml plastic pots and provided with 40% sucrose solution until they could be transferred to a –80°C freezer at the end of each tracking day, to be analysed later for their disease status and, if diseased, pathogen load.

### Data Analysis

Based on the recorded radar signals, tracks were manually digitalized using a custom-made TAS - Track Analysis Software V1.0 (by Shane Hatty, Rothamsted Research 2008). Positional information was transformed from polar coordinates (range, angle) to Cartesian coordinates (x, y) for further analysis.

From the tracks we extracted two types of parameter using Matlab V7 (www.mathworks.com): 1) parameters related to the actual flight characteristics of the bee irrespective of its homing success (hereafter Flight parameter: mean and maximum flight speed, mean and maximum acceleration, track straightness with reference to the last track coordinate, mean and maximum turning angle, proportion of flight time of total track duration, and number of stops (defined as tracking discontinuities longer than 10 seconds and with a distance between last recorded position and position of re-emergence smaller than 10 m) and 2) parameters characterizing the spatial and temporal dimensions of the track itself (hereafter Track parameter: track length, track duration (i.e. homing time), total flight time, directionality towards the hive measures as both “Approachiness” and bearing angles after half of the homing distance (see below and file S1 for further details)).

As track parameters, in contrast to flight parameters, are affected by homing success, we only compared track parameters between treatment groups for bees that successfully returned to the colony. Total and mean stop duration qualified as both track and flight parameters and were therefore included in the analysis of both parameters types.

**Figure 3:**
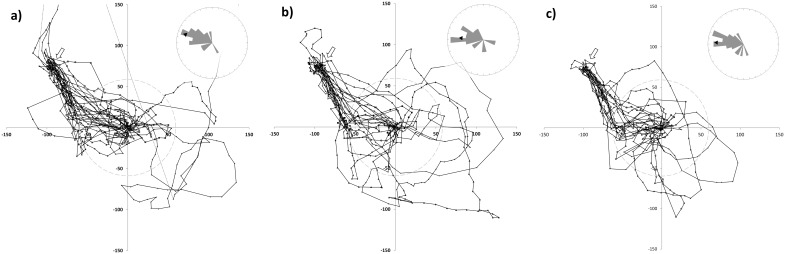
Flight paths of returned bees. Graphical representation of all recorded flight paths of successfully homed bees: a) sucrose-control bees (C_S_), b) gut-extract-control bees (C_GE_) and c) *Nosema*-inoculated (Nc). The release site is located at the origin of the axes and the hive position indicated by the white arrow. The grey circle illustrates the 60 m perimeter at which track heading direction was assessed and which is illustrated in the inserts (black arrow: overall mean heading direction).

**Figure 4:**
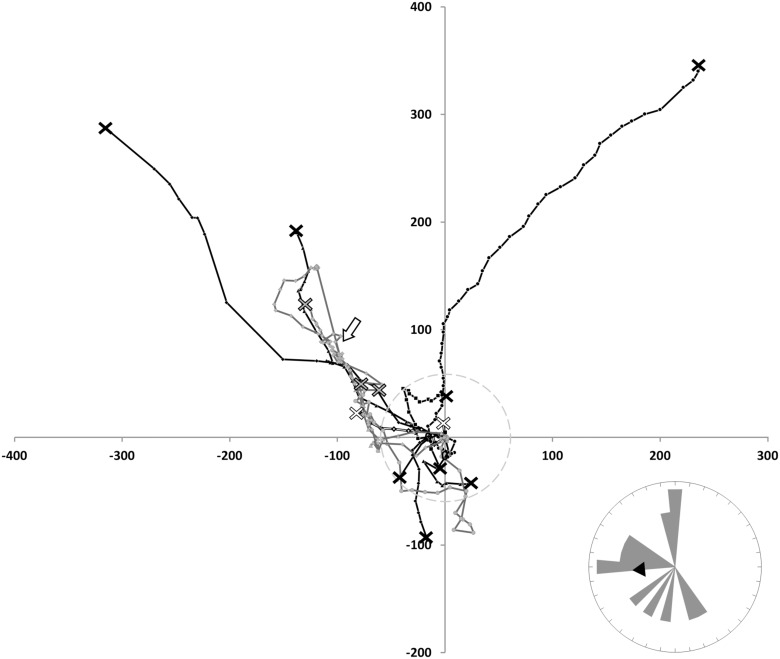
Flight path of non-returned bees. Graphical representation of all recorded flight paths of non-retuning bees and the spatial distribution of the final bee positions (C_S_: double black line, white diamonds, end-position = white cross; C_GE_: grey line, light grey markers, end-position = grey cross; Nc: black line, black markers, end-position = black cross). The release site is located at the origin of the axes and the hive position indicated by the white arrow. The grey circle illustrates the 60 m perimeter at which track heading direction was assessed and which is illustrated in the inserts (black arrow: overall mean heading direction).

**Figure 5:**
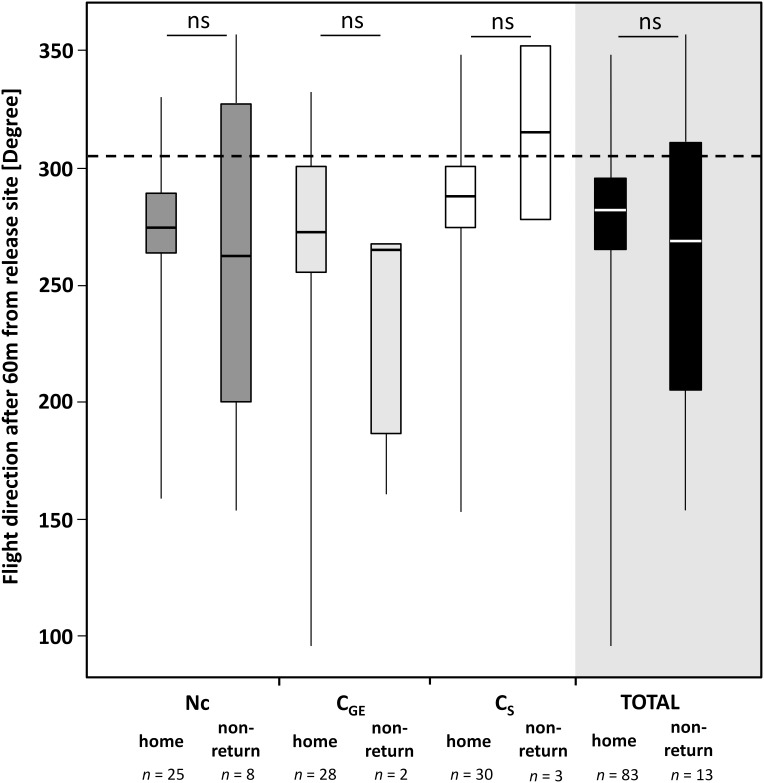
Flight direction. Box plots (box: median (central line) ± quartiles; whiskers: minimum – maximum values) of heading direction of homed and non-returning bees within each treatment (Nc = *Nosema*-inoculated, C_GE_ = bee gut-extract-control, C_S_ = sucrose-control) group after crossing the 60 m perimeter around the release site. The direction of the hive is indicated by the central dashed line. As the majority of the bees followed the available landmarks rather than assuming a direct approach to the hive, heading directions after 60 m from the release site generally deviate from the ideal direction. Using a linear mixed model (LMM) fitted by restricted maximum likelihood (REML), there was neither a statistically significant difference between homed and non-returning bees nor among the treatment groups.

In a recent homing-experiment using Radar tracking, Fischer et al. (2014) [51] used bearing angles to assess navigational performance of homing bees. After displacement pre-trained bees typically exhibited a two-component flight path composed of a vector flight (the established flight route prior to replacement) and a homing flight after re-orientation [51]. In contrast, the foragers in this study were freely foraging in the landscape exhibiting homing flights comparable to the homing-component of Fischer et al. (2014) [51]. We thus analysed the heading direction (Figs. 3–5) when the bee fist flew beyond 60 m from the release site (half of the homing distance) or at the last known position in cases where the tracks did not progress beyond 60 m (*n* = 5). This approach allowed a comparison of the spatial distribution of returning and non-returning bees on the homing flight accounting for initial re-orientation loops.

Additionally we assessed the navigational abilities of the tracked bees through the goal-directedness of the entire homing flights towards the hive (goal). We used mean goal directedness of the entire track (hereafter referred to as “Approachiness” A) based on the angle (α) formed between a bee’s position (t_n_), the position of the hive and the next recorded bee position (t_n+1_). Using the difference of maximal and realized angle divided by the maximal angle for every consecutive pair coordinates A results in A = 1 (α = 0°) for a direct approach, A =  −1 (α = 180°) for flights away from the hive and A = 0 (α = maximal for the respective distance between the two focal positions of the bee) for equidistant orbiting flights. The individual A-values were multiplied by the percentage of the total track length that was covered between the two positions (t_n_ to t_n+1_) to adjust for potentially different contributions of each track-section to the overall bee movement. The mean of all distance-adjusted A-values per track were used as measure for flight directionality.

We also tested the utilization of available landmarks by the bees as a proximate measure of orientation ability. We compared the actual flight paths to two hypothetical homing flight scenarios: S1) the direct (straight) flight from the release site (RS) to the hive, and S2) a flight along the available landmarks following the field margin connecting release site to hive (Fig. 1b). For both hypothetical paths, we calculated the coordinates of 100 equidistantly spaced markers representing the path. Using the coordinates of the bee’s position during the homing flight, we determined the distance to the nearest possible marker under either scenario. Both shortest total and mean distances (i.e. minimum deviation from either S1 or S2) of a bee’s flight under either scenario were used to assign the realized flight to one of these two hypothetical flight paths. During the initial orientation loops the nearest reference points to either scenario were typically centred on the release site were both hypothetical flight path differ only marginally (Figs. 1 and 3, 4) and assignment to either scenario was only possible once the bees embarked on the homebound flight. The vast majority of the directional homing flights following the orientation loop(s) was observed within close proximity to the hypothetical flight paths (Figs. 1 and 3) allowing for a representative matching of the flight paths of the different treatment groups to either hypothetical flight path.

### Statistics

All statistical analyses were performed using the statistical software GenStat V14.1. (VSN International, 2011).

Radar-inferred parameters were compared among treatment groups using a linear mixed model (LMM) fitted by restricted maximum likelihood (REML), with a crossed fixed model (treatment × homing success) and a nested random model (tracking day × bee). Where necessary, parameters were transformed to achieve homogeneity of variances and normality. All results are reported as predicted or back-transformed (geometric) means with 95% confidence intervals. However, these individual analyses did not account for potential interactions among the recorded parameters and the magnitude and direction such interactions might have on the different treatment groups. Thus, to complement LMM analyses we used normalized and standardized track data to separately analyse pooled track parameters, pooled flight parameters and the full set of parameters using Canonical Variate Analysis (CVA).

The effects of ambient temperature, handling time and spore load on track and flight parameters were assessed for each treatment group separately using simple linear regression (SLR) on normalized data. The separation of treatment groups allowed assessment of the effects of a variable within a treatment group, overcoming the confounding effects of the treatment *per se*, which were appropriately analysed through the LMM-analyses. The effects of infection intensity (spore load) on bee flights were undertaken for Nc-bees only, as only these were found to be infected with *Nosema* spores.

## Results

### Experimental Bees

Over the course of the experiment we introduced a total of 2030 bees to the host colony (HC), of which 713 were inoculated with *N. ceranae* (Nc). The 1317 control bees were treated with either *Nosema*-free gut extract (C_GE_: 624) or 40% sucrose (C_S_: 693). As bees of all three treatment groups originated from a common brood-frame and developed within the same colony environment, the age groups of these bees was assumed to be similar across treatments.

All tests of *Nosema* species composition in the inocula showed the exclusive presence of *N. ceranae*. Likewise both control inocula were confirmed to be *Nosema*-negative. Overall we tested the homing ability of 105 bees (Nc: 38, C_GE_: 32, C_S_: 35). Of these, we successfully recorded flight tracks of 33 *Nosema*-inoculated bees (Nc), 30 C_GE_ bees and 33 C_S_ bees. These tracks either ended at the colony for successfully homing bees, or were truncated suggesting that the bee stopped flying before reaching the hive.

### Handling Time

The mean handling times (mean ± SD) per treatment group were Nc: 322s±128s (range: 142s–699s), C_GE_: 300s±104s (range: 158s–573s) and C_S_: 322s±122s (range: 148s–590s) and did not statistically differ among treatment groups (Kruskal-Wallis ANOVA: *H* = 0.223, *d.f.*  = 2, *p* = 0.894). We found no significant effect of handling time on any of the flight or track parameters, even after excluding data points identified through Cook’s influence statistics (*C_i_*,[58]) as strongly affecting the overall outcome (see file S1).

### Spore Load

All tracked bees that returned to the hive and could be captured on their return (76 bees (Nc: 23; C_GE_: 26; C_S_: 27)) were screened for their *Nosema* load. *Nosema* spores were exclusively found in the mid-guts of bees from the inoculated Nc-group, while both control groups were *Nosema* free, confirming both successful spore inoculation and no cross-contamination after the application of the treatments.

Within the Nc-group, spore loads varied from 2.37×10^6^ to 4.70×10^7^ spores per midgut, with a mean spore load of 1.82×10^7^ (±9.64×10^6^: SD), which compares well to the spore loads of bees in naturally infected colonies [25].

### Homing Success

Of the 105 tested bees, 87 (82.9%) successfully returned to the colony. Homing success significantly differed between the *Nosema*-inoculated group (Nc), of which 13 of 38 (34.2%) did not return to the hive, and the *Nosema*-free control bees (*Χ^2^* = 12.3, *d.f.*  = 2, *p* = 0.002), of which consistently no more than 10% (C_GE_: 3 of 32, C_S_: 2 of 35; *Χ^2^* = 0.13, *d.f.*  = 1, *p* = 0.72) failed to return home (Fig. 2).

Of the 105 tested individuals, we were only able to successfully radar track 96 due to technical errors during recording. In total, eighty-three of these 96 bees (86.5%) were homing bees, reducing the overall number of non-returning bees whose initial flights could be recorded to 8, 3 and 2, Nc, C_GE_ and C_S_ respectively. Examining the pre-release conditions, we found no significant differences in the handling times of bees that failed to return (306s±120s (range: 150s–600s)) and those that successfully returned to the hive (315s±119s (range: 142s–699s)) (*H* = 0.122, *d.f.*  = 1, *p* = 0.73). Likewise there was no significant difference in the ambient temperatures during the homing flights of non-returning and homed bees; temperatures ranged from 12.5°C to 22.0°C (T_mean_ = 17.3°C) and 13.0°C to 22.0°C (T_mean_ = 16.6°C), respectively (*U*_19, 87_ = 640.5, *p* = 0.127).

### Homing Time

Homing time (HT), i.e. track duration of returning bees, in all three treatment groups showed considerable variation, ranging from less than one minute up to well over 30 min (Table 1), but did not vary between treatments (*F^HT^_2, 71.5_* = 0.85, *p*<0.433). On average, bees in the different treatment groups required 184 s (C.I.: 118, 288), 196 s (C.I.: 131, 292) and 227 s (C.I.: 149, 344) for Nc, C_S_ and C_GE_, respectively, to return to the colony.

**Table 1:** Summary of flight parameters.

	treatment	N.c.			C_GE_			C_S_			total			LMM (REML)-variance component analyses		
		mean	−95% C.I.	+95% C.I.	mean	−95% C.I.	+95% C.I.	mean	−95% C.I.	+95% C.I.	mean	−95% C.I.	+95% C.I.	total home vs. total lost		
**home**														*F*	*d.d.f. (n.d.f. = 1)*	*p*
	**sample size**	**25**			**28**			**30**			**83**					
	track length [m]*	**249.00**	201.40	307.90	**250.10**	205.50	304.30	**225.40**	186.60	272.30	-			**-**		
	homing time [s]*	**105.89**	75.41	148.70	**90.44**	65.85	124.20	**88.10**	64.43	120.50	-			**-**		
	**total flight time [s]**	**101.05**	73.87	138.20	**84.38**	63.77	111.70	**87.49**	65.55	116.80	**90.70**	73.59	111.78	12.27	89.8	***<0.01***
	**continual flight time [s]**	**36.50**	25.99	51.22	**32.28**	23.49	44.36	**28.47**	21.12	38.36	**32.24**	26.82	38.76	8.48	51.3	***<0.01***
	mean stop time [s]**	**22.48**	8.99	54.2	**36.54**	15.21	85.90	**20.43**	8.88	45.5	**25.6**	15.03	43.26	0.33	83.2	*0.57*
	total stop time [s]**	**31.95**	11.95	32.80	**59.25**	22.68	152.30	**29.01**	11.80	69.40	**38.06**	22.13	65.00	0.48	86.0	*0.49*
	**continual stop time [s]**	**41.38**	26.94	63.60	**70.17**	46.74	105.30	**76.49**	51.73	113.10	**60.56**	46.62	78.70	6.55	112.6	***0.01***
	**mean number of stops**	**1.24**	0.79	1.80	**1.43**	0.96	2.00	**1.12**	0.73	1.59	**1.26**	0.99	1.55	0.01	90.0	*0.94*
	**continual flight distance [m]**	**72.78**	47.23	112.20	**70.89**	47.33	106.20	**51.62**	35.34	75.40	**64.34**	50.90	81.32	8.42	48.7	***<0.01***
	**mean flight speed [ms^−1^]**	**2.98**	2.55	3.42	**3.42**	3.02	3.83	**3.18**	2.78	3.57	**3.19**	2.86	3.53	0.16	89.6	*0.69*
	**max flight speed [ms^−1^]**	**7.21**	6.35	8.19	**7.28**	6.49	8.17	**7.72**	6.87	8.69	**7.40**	6.75	8.12	0.52	89.9	*0.47*
	**mean acceleration [ms^−2^]**	**0.98**	0.83	1.13	**1.16**	1.02	1.30	**1.06**	0.92	1.19	**1.07**	0.96	1.17	0.04	90.0	*0.84*
	**max acceleration [ms^−2^]**	**3.22**	2.75	3.76	**3.40**	2.96	3.91	**3.68**	3.18	4.26	**2.56**	1.96	3.38	1.00	86.6	*0.32*
	**approachiness**	**0.37**	0.29	0.46	**0.40**	0.33	0.48	**0.43**	0.36	0.50	**0.40**	0.35	0.45	39.82	89.5	***<0.001***
	track straightness*	**0.52**	0.43	0.62	**0.55**	0.47	0.65	**0.59**	0.51	0.68	**0.55**	0.50	0.61	**-**		
**non-returning**																
	**sample size**	**8**			**2**			**3**			**13**					
	track length [m]*	**-**			**-**			**-**			-					
	homing time [s]*	**-**			**-**			**-**			-					
	**total flight time [s]**	**47.47**	28.85	78.10	**9.82**	3.76	25.70	**131.68**	49.94	347.20	**39.45**	24.05	64.72			
	**continual flight time [s]**	**13.68**	8.06	23.23	**27.81**	12.90	59.94	**9.64**	1.91	48.58	**15.42**	8.28	28.74			
	mean stop time [s]**	**52.41**	11.35	230.00	**64.72**	2.60	1199.90	**5.46**	−0.65	116.80	**27.30**	5.56	121.03			
	total stop time [s]**	**107.61**	19.85	564.80	**83.81**	2.12	2300.70	**6.07**	−0.74	190.90	**39.23**	6.72	280.60			
	**continual stop time [s]**	**120.42**	61.01	237.70	**116.18**	48.46	278.50	**44.50**	3.56	556.80	**85.38**	33.78	215.8			
	**mean number of stops**	**1.59**	0.74	2.86	**1.45**	0.11	4.42	**0.41**	−0.36	2.13	**1.08**	0.39	2.09			
	**continual flight distance [m]**	**23.48**	12.02	45.90	**37.41**	14.38	97.30	**37.63**	4.65	304.40	**32.09**	14.45	71.30			
	**mean flight speed [ms^−1^]**	**3.14**	2.51	3.76	**2.54**	1.35	3.73	**3.01**	1.86	4.16	**2.90**	2.27	3.52			
	**max flight speed [ms^−1^]**	**7.87**	6.50	9.53	**5.31**	3.71	7.60	**5.82**	4.04	8.40	**6.24**	5.17	7.55			
	**mean acceleration [ms^−2^]**	**1.07**	0.84	1.30	**0.79**	0.34	1.24	**1.05**	0.61	1.49	**0.97**	0.74	1.20			
	**max acceleration [ms^−2^]**	**3.56**	2.72	4.66	**2.13**	1.25	3.64	**2.23**	1.30	3.80	**3.43**	3.13	3.75			
	**approachiness**	−**0.16**	−0.29	−0.03	**0.20**	−0.05	0.46	**0.40**	0.15	0.66	**0.15**	0.02	0.28			
**total**														**total N.c. vs. total C**_GE_ vs. total C_S_		
	**sample size**	**33**			**30**			**33**						*F*	*d.d.f. (n.d.f. = 2)*	*p*
	track length [m]*	**249.00**	201.40	307.90	**250.10**	205.50	304.30	**225.40**	186.60	272.30				*0.30*	*77.7*	0.74*
	homing time [s]*	**105.89**	75.41	148.70	**90.44**	65.85	124.20	**88.10**	64.43	120.50				*0.28*	*76.4*	0.75*
	**total flight time [s]**	**69.26**	50.85	94.30	**28.79**	17.30	47.90	**107.34**	64.37	179.00				0.73	85.0	*0.48*
	**continual flight time [s]**	**22.34**	16.32	30.59	**29.96**	19.77	45.40	**16.57**	7.28	37.70				0.30	70.1	*0.75*
	mean stop time [s]**	**34.41**	14.02	82.50	**48.67**	9.90	225.30	**10.77**	1.60	52.20				0.36	83.2	*0.55*
	total stop time [s]**	**58.82**	22.18	153.40	**70.48**	11.86	396.30	**13.57**	1.65	79.10				0.87	86.0	*0.42*
	**continual stop time [s]**	**70.59**	46.78	106.50	**90.29**	55.41	147.10	**58.34**	16.20	210.10				1.08	147.1	*0.34*
	**mean number of stops**	**1.41**	0.92	2.03	**1.44**	0.62	2.68	**0.73**	0.15	1.60				0.69	90.0	*0.50*
	**continual flight distance [m]**	**41.34**	27.75	61.58	**51.50**	30.65	86.54	**44.07**	15.23	127.51				0.45	68.6	*0.64*
	**mean flight speed [ms^−1^]**	**3.06**	2.63	3.49	**2.98**	2.34	3.63	**3.09**	2.45	3.73				0.68	89.6	*0.41*
	**max flight speed [ms^−1^]**	**7.53**	6.65	8.53	**6.22**	5.12	7.56	**6.71**	5.51	8.16				0.49	89.9	*0.49*
	**mean acceleration [ms^−2^]**	**1.02**	0.88	1.17	**0.97**	0.74	1.21	**1.05**	0.82	1.30				1.35	85.3	*0.27*
	**max acceleration [ms^−2^]**	**3.39**	2.89	3.96	**2.69**	2.04	3.54	**2.86**	2.17	3.78				0.38	88.2	*0.68*
	**approachiness**	**0.11**	0.03	0.19	**0.30**	0.17	0.44	**0.42**	0.28	0.55				9.98	86.3	***0.001***
	track straightness*	**0.52**	0.43	0.62	**0.55**	0.47	0.65	**0.59**	0.51	0.68				*0.60*	*77.1*	0.55*

Table of sample sizes, predicted means and 95% confidence intervals (C.I.) of the analysed flight characteristics, and statistics for homed bees, non-returning bees and the total dataset. Parameters were compared using linear mixed models (LMM) fitted by restricted maximum likelihood (REML), with a crossed fixed model (treatment × homing success) and a nested random model (tracking day × bee). Denominator degrees of freedom (*d.d.f.*) are reported individually whereas the test-wide unvaried nominator degree of freedom (*n.d.f.*) is given in the heading. Parameters marked with an asterisk (*) were only for successfully homed bees (see also Table S1). Parameters marked with two asterisks (**) were assessed excluding composite tracks (*n* = 3) consisting of two sub-tracks recorded over a long period of time i.e. putative non-returning bees re-emerging on the radar screen after a long period of discontinuous radar-tracking (tracking of other bees interspersed with non-tracking times while bees were prepared and displaced), characterized by one very long putative stop separating the two track-components.

### Flight and Track Characteristics

**Figure 6:**
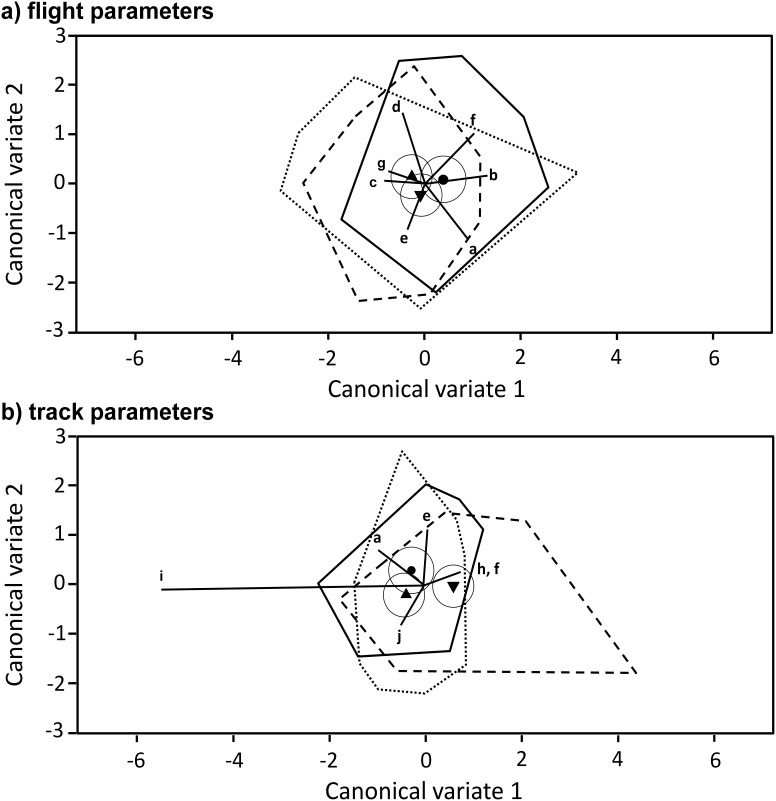
Comparison of flight performance. Biplots of a Canonical Variate Analysis (CVA) of a) normalized and standardized flight parameters (a: max. acceleration, b: mean acceleration, c: max. velocity, d: mean velocity, e: mean gap duration, f: total gap-duration, g: straightness) and b) normalized and standardized track parameters (h: actual total flight-time, i: Approachiness, j: total track duration, k: track length, f: total gap duration, e: mean gap duration; h and f coincident). Treatment mean scores+95% C.I. (solid circles) and circumferential polygons are given as follows: sucrose-control C_S_: ▴, dotted line; gut-extract control C_GE_: •, solid line; *Nosema*-inoculated Nc: ▾, dashed line. Both graphs show a low discriminative power of both types of parameter in separating *Nosema*-infected and uninfected bees, with only Approachiness (i), highly affected by homing success, allowing some separation of the infected non-returning bees from the rest.

Overall, flight characteristics varied widely within treatment groups (TR) and none of the parameters differed significantly between the *Nosema* infected bees and the control bees (Table 1) even if returning and non-returning bees were analysed separately (Table S1). This indicates that an infection with *N. ceranae* does not affect the characteristics of the flight itself. Analysing all flight parameters together using a multivariate approach (Canonical Variate Analysis (CVA)) confirmed that flight parameters do not allow a clear separation of the treatment groups (Fig. 6a).

For the track parameters, we found a similar pattern. Approachiness (a measure of directionality of hive approach) was the only parameter that showed a highly significant reduction from 0.30 and 0.42, for C_GE_ and C_S_ bees respectively, to 0.11 in the Nc-group (*F^TR^_2, 86.3_* = 9.98, *p*<0.001) (Tables 1 and S1). This effect is expected as the overall Approachiness value increases towards the end of a successful homing flight and thus we expect reduced values for non-returning bees, which were heavily represented in the Nc-group. In comparison with all other parameters, only “Approachiness” and actual total flight time showed some separation of the Nc-group from the controls (Fig. 6b). Both of these parameters are greatly affected by homing success.

### Track Continuity

To assess flight energetics, we measured i) the number, total duration and mean duration of flight interruptions (stops) and ii) interrupted flights, as well as continuous, uninterrupted flight time and corresponding distances (Table 1). Again, all of these parameters showed high variation independent of treatment and were not significantly different among treatments (Table 1).

Non-returning bees were characteristically different from returning bees. They exhibited a reduced continuous flight time (*F^HS^*_1, 143.8_ = 8.94, *p* = 0.003) and distance between “stops” (*F^HS^_1, 130.6_* = 8.55, *p* = 0.004) and an increase in continuous stop duration (*F^HS^_1, 115.0_* = 4.50, *p* = 0.036) compared to homed bees (Table 1).

### Navigational Abilities

We found that a significant majority (Nc: 65.6%, C_GE_: 70.0%, C_S_: 63.6%, total: 66.3%) of experimentally displaced bees more closely followed the available landmarks back to their hive (S2 - flights) rather than using a direct route (S1 - flights) (*Χ^2^* = 10.12, *d.f.*  = 1, *p* = 0.0015), with no significant difference in the frequency of S1–flights and S2–flights between the treatment groups (*Χ^2^* = 0.295, *d.f.*  = 2, *p* = 0.86).

Successfully homing bees, independent of treatment group, predominantly took a flight path with mean heading directions at 60m from the release site of 284.3° ±34.4° for the C_S_-control, 259.2° ±59.0° for the C_GE_-control and 268.8° ±36.9° for the *Nosema*-inoculated bees. The heading directions of the non-returning bees were 315.2° ±52.3°(C_S_), 231.3° ±61.3°(C_GE_) and 261.3° ±79.5°(Nc). This is in line with our findings on the utilized flight paths as this bearing angle matches the route of the field track represented in the first part of flight scenario S2 (Fig. 1). Neither treatment (TR), homing success (HS) nor the interaction of both (TR*HS) revealed differences in the mean heading direction (*F^TR^_2, 89.0_* = 3.05, *p*>0.05; *F^HS^_1, 89.0_* = 0.16, *p*>0.69; F^TR^*^HS^_2, 89.0_ = 0.83, *p*>0.44) (Figs. 3–5).

Of the non-returning bees, the majority flew approximately along the landmarks (e.g. successfully returning bees) but did not arrive at the colony either because the flight path was truncated before the colony was reached or because the bee overshot along the hedgerow, and did not manage to return to the hive afterwards. With the exception of one bee assuming a long and straight flight path in the wrong direction (and leaving the range of the radar, not to return), all bees that did not follow landmarks immediately assumed flight paths that were interpreted as the initial part of an orientation loop, as seen in successfully homed bees. Thus, apart from one bee, the track information of the non-returning bees provides no clear signs of disorientation (Fig. 4).

### Effects of Infection Intensity

Though we found no significant differences in flight and track parameters between the inoculated bees and the control bees (see above), we also tested whether spore load might explain within-treatment variation in flight parameters. Using simple linear regression (SLR) on *Nosema*-inoculated bees (Nc-group), we found no significant relationship between infection intensity (measured as both spores per mid-gut and spore density (spores/µl)) and the flight and track parameters. However, there was a positive, yet non-significant, trend between normalized spore load and both mean and total stop duration (spore density vs. mean stop duration/total stop duration: *d.f*. _regression, total_ = 1, 20/1, 20; *s.s.*  = 1.661/2.28; *v.r.*  = 3.55/3.33; *p* = 0.075/0.084) (Fig. S1), suggesting these relationships warrant closer attention in future studies.

## Discussion

The performance of a honeybee colony is governed by the performance of its members. Changes to the foraging behaviour and success of its individuals may compromise the colony as a whole. Likewise such changes may have important implications for other intra- and inter-specific interactions. Looking at the effects of an emerging bee pathogen, *Nosema ceranae*, on homing performance of foraging bees, we aimed to gain a better understanding of how this invasive pathogen affects the behaviour of individual bees and may ultimately shape the performance of the entire colony, and of how this pathogen might enhance its own transmission.

In line with previous reports (e.g. [31]), we find that the homing abilities of *Nosema*-inoculated bees are strongly compromised. The proportion of non-returning bees is quantitatively similar to that detected by Kralj & Fuchs (2010) [31], who reported a loss of approximately 19% of infected bees compared to 7% of a clean control group, at a homing distance of only 30 m, which is very short compared to even conservative estimates of honeybee foraging distances (e.g. mean foraging distance: 1000m (May), 5500m (August) [59]; mean: 1543m (range: 62–10,000m) [60]). Testing returning foragers on a 120 m homing flight, as we carried out here, is likely to provide a more realistic estimate of *Nosema*-induced losses in foragers.

The striking effect on homing success was, surprisingly, not reflected in the flight characteristics that we measured. Of the bees that returned home, we did not find any differences in the time required to return to the colony, in contrast to the findings of Kralj & Fuchs (2010) [31]. The latter study released and compared return times of bees only in discrete pairs and did not account for within group variability across the experiment, thus considerably reducing the variability of their data set. In our study, even after statistically accounting for daily and individual variation, homing times varied greatly among and within treatment groups, though neither handling times nor ambient temperature had a significant effect on homing time.

*Nosema apis* may accelerate the age-polyethism of bees, i.e. bees have an earlier onset of foraging and age more quickly [1]. *N. ceranae* is likely to cause similar effects. Though not experimentally controlled, tracking day (i.e. the bee’s physical and behavioural age) as a source of variation was taken into account in the statistical analyses. As the experiment was conducted over several weeks, all three treatment groups comprised a widely overlapping range of differently experienced and aged foragers. The overall effects on homing performance may be differentially affected by both experience and old age, and are unlikely to be solely caused by an accelerated behavioural schedule. However, as our observations on flight performance are restricted to *Nosema* infected bees that developed into foragers we may underestimate the colony-level effects of *Nosema* on honeybees as *Nosema* infection may cause some bees not even to commence foraging.

Detailed analysis of the flight parameters measured by the radar likewise revealed no differences in performance among treatments. *Nosema*-inoculated bees neither exhibited reduced flight speed, acceleration, continuous flight times, continuously covered flight distances nor increased numbers of stops or stopping times, as compared to control groups. Apart from a high proportion of bees failing to return home (Fig. 4), our data show no clear effect of *N. ceranae* on a bee’s flight performance, even for infection levels as high as 46.9×10^6^ spores per midgut.

Kralj & Fuchs (2010) [31] showed significant disorientation of *Nosema*-infected bees when challenged with a short-range displace-release-entrance choice experiment (6–10 m). They suggested these orientation deficits contribute to the reduction in homing success and an increased disease spread to adjacent colonies through the drift of infected workers to those colonies. Our analyses of the flight patterns and heading directions on 120 m homing flights revealed no pernicious effect of *N. ceranae* on a bees’ ability to utilize available landmarks for orientation in a real world setting. Several of the non-returning bees actually appeared to follow the landmarks but did not manage to home in on the colony. The challenge of distinguishing an unexpected dummy entrance from the real colony entrance, as opposed to the challenge of utilizing known landmarks to re-orientate towards the hive, might explain the discrepancy in the results of Kralj & Fuchs (2010) [31] and this study.

Two thirds of the bees from each treatment group were clearly navigating along field margins typical for honeybees (e.g. [61], [62]) (Figs. 3 and 4). The remarkable consistency of this pattern despite the significantly different representation of non-returning bees among the groups suggests a minor, if any, effect of *N. ceranae* on the orientation abilities of foragers. This is supported by the fact that all but one (*n* = 13) non-returning bee showed flight tracks well matching the initial parts of the flight paths observed in successfully homed bees.

The significant reduction in directionality of the approach to the colony (Approachiness) of infected bees, which at first glance indicated an impaired orientation, can be explained by parameter dependency on homing success, with non-returning bees typically achieving low Approachiness scores. When taking homing success into account, no differences were found. Likewise, both homed and non-returning bees did not differ significantly in the overall heading direction, after covering 60 m from the release site.

With no clear evidence of *Nosema* affecting flight characteristics and navigational abilities, *Nosema*-induced energetic limitation [32], [33] provides a plausible, alternative explanation for the observed losses of infected bees in the field. Honeybee flight is energetically costly and is fuelled by relatively low energy reserves. The increased energetic demands imposed by *Nosema* on bees [32] could compromise a bee’s ability to compensate for small displacements on the returning flight. The extended flight burden imposed by our experimental displacement of 120 m, a detour likely to occur naturally through wind drift, was apparently sufficient to impact on homing success of the *Nosema*-infected bees.

For the non-returning bees, which were mostly inoculated, we found a significant reduction of continuously sustained flight times and vastly increased stop durations the latter being also weakly associated with infection intensity in successfully returned bees (Fig. S1). Though we could not test the infection levels of non-returning bees, it is reasonable to assume that at least all non-returning bees from the Nc - group were infected, given that all returning bees from this group were infected with *Nosema* (spore loads from 7.9×10^3^ to 1.57×10^5^ spores/µl).

In addition to *Nosema* infected bees being unable to return to the colony, possible increased energetic demands in foragers through *Nosema* infection may have more far-reaching effects for the colony. Honeybee foraging is energetically optimized by a trade off between energy delivery and associated expenditure costs, thus maximizing energy efficiency [63]. Increases in costs may lead to inefficient foraging, either through reducing crop loads in order to minimize flight costs (thus not adequately exploiting available resources), or over-investing in the transport of a normal crop-load. Though this remains to be studied in detail, both scenarios would reduce the net energy influx into the colony, therefore lowering the resources crucial for colony growth. Also a reduced willingness of *Nosema*-infected bees to share food within the hive, as demonstrated by Naug & Gibbs (2009) [33], may contribute to an energetic deprivation of the colony, causing pronounced colony level effects of *Nosema*.

Though the energetics of the bees was not tested in this study, our data can be plausibly explained by energetic stress imposed by a *Nosema* infection. However, the lack of a clear difference between treatment groups in flight performance and the considerable spore loads found in some successfully returning bees of the Nc-treatment group also indicate that the interactions between the host and the parasite may be more complex, and may not readily affect host bee flight behaviour.

By limiting the probability of an infected bee returning to its hive, *Nosema* may limit its spread within the host bee’s colony but promote its spread to con-specific and hetero-specific hosts because pathogens may be transmitted by foragers on shared floral resources [64], [65]. However, the behavioural effects that we detected seem, if anything, to limit the further spread of *Nosema* spores between colonies and heterospecific hosts; though our data suggest that infected bees fail to return to their host colony after experimental displacement, such bees seems to be forced to permanently stop on their path back to the colony, and may be therefore unlikely to transmit spores. Further studies of the foraging behaviour of infected and non-returning bees is needed to test determine their role in potentially enhancing the transmission of the parasite.

In summary, our study shows that *Nosema-*induced homing deficits on displaced bees are mostly expressed in reduced flight performance rather than through a compromised navigation. As suggested by truncated flight paths, *Nosema*-infected bees seem to be challenged by sustained periods of flight, leading to increased mortality of bees *en route* to their home colony. Beyond these pernicious effects for the host, this has marked implications for the pathogen. Whereas navigational deficits may potentially facilitate the spread of spores to new host colonies through drifting, bees physically incapable of reaching any hive on a return flight from foraging grounds might also compromise the survival of the pathogen. This would suggest a non-directional host-response to the infection rather than an active behavioural manipulation by *Nosema* to facilitate its transmission. Further research is needed to explore both the details of the trade-off between these effects on the host and the pathogen, especially in commercial apiaries with high colony densities, and the underlying mechanisms (physiological constraints, navigational deficits) causing the behavioural effects in *N. ceranae* infected bees.

## Supporting Information

Figure S1 **Correlation of pathogen-load and stop-duration.** Simple linear regression plots and 95% confidence limits of a bee’s log-mean and total stop duration (s) and spore load (spores/µl) for *Nosema*-infected bees successively excluding those data points (a, b), which have been identified as having a disproportionately strong effect on the overall regression by Cook-statistics. Though non-significant for the full data set, the exclusion of either one or both of the outliers returned a significant positive relationship between stop time and *Nosema*-level within the infected group. The predictive power of spore load increased from around 10% (full data set) to a maximum of over 40% (both outliers excluded).
(TIF)

Table S1 **Summary of flight parameters for home vs. non-returning bees.** Table of predicted means and 95% confidence intervals (C.I.) of the analysed flight characteristics for both homing and non-returning bees per treatment group (C_S_ = sucrose-control, C_GE_ = bee gut-extract-control, Nc = *Nosema*-inoculated). Within each homing group (home, non-returning) parameters were compared between treatment groups using linear mixed models (LMM) fitted by restricted maximum likelihood (REML), with treatment as fixed model and a nested random model (tracking day × bee). Denominator degrees of freedom (*d.d.f.*) are reported individually whereas the test-wide unvaried nominator degree of freedom (*n.d.f.*) is given in the heading. Due to data specific characteristics the p-value labelled with an asterisk (*) is given as Wald-statistic (Wald-statistic; *d.f*.; *Chi*-probability) calculated from the LMM (REML). As straightness of the track is calculated from the shortest vs. the realized flight path from the release site to the hive, this parameter could not be analysed for non-returning bees.
(DOCX)

File S1 **Supporting information.** Detailed description of the experimental prodedures, data analyses and additional statistical analyses of the results.
(DOCX)
