# Supplementary material for: So Near and Yet So Far: Harmonic Radar Reveals Reduced Homing Ability of Nosema Infected Honeybees
Source: PLoS One. 2014 Aug 6;9(8):e103989. doi: 10.1371/journal.pone.0103989 (PMC4123971; doi:10.1371/journal.pone.0103989)
Supplement: Table S1 — Summary of flight parameters for home vs. non-returning bees. Table of predicted means and 95% confidence intervals (C.I.) of the analysed flight characteristics for both homing and non-returning bees per treatment group (CS = sucrose-control, CGE = bee gut-extract-control, Nc = Nosema-inoculated). Within each homing group (home, non-returning) parameters were compared between treatment groups using linear mixed models (LMM) fitted by restricted maximum likelihood (REML), with treatment as fixed model and a nested random model (tracking day × bee). Denominator degrees of freedom (d.d.f.) are reported individually whereas the test-wide unvaried nominator degree of freedom (n.d.f.) is given in the heading. Due to data specific characteristics the p-value labelled with an asterisk (*) is given as Wald-statistic (Wald-statistic; d.f.; Chi-probability) calculated from the LMM (REML). As straightness of the track is calculated from the shortest vs. the realized flight path from the release site to the hive, this parameter could not be analysed for non-returning bees. (DOCX) [file pone.0103989.s002.docx]

**Table S1:**

| **parameter** |  | **home** | | | | | |  | **non-returned** | | | | | |
| --- | --- | --- | --- | --- | --- | --- | --- | --- | --- | --- | --- | --- | --- | --- |
|  |  | **C_S_** | **C_GE_** | **Nc** | ***F*** | ***d.d.f. (n.d.f.* = 2*)*** | ***p*** |  | **C_S_** | **C_GE_** | **Nc** | ***F*** | ***d.d.f. (n.d.f.* = 2*)*** | ***p*** |
| **track length [m]** |  | 224.8 | 239.7 | 248 | *0.30* | *77.7* | *0.74* |  | 53.9 | 375.2 | 158.7 | *3.29* | *10.0* | *0.08* |
|  |  | (188.3 / 268.5) | (198.5 / 289.5) | (203.3 / 302.6) |  |  |  |  | (17.1 / 170.3) | (146.7 / 959.8) | (89.3 / 282.1) |  |  |  |
| **total track duration [s]** |  | 196.0 | 226.5 | 184.6 | *0.28* | *76.4* | *0.75* |  | 40.1 | 802.3 | 372.3 | *2.53* | *10.0* | *0.13* |
|  |  | ( 131.7 / 291.5) | (149.4 / 343.5) | (118.2 / 288.3) |  |  |  |  | (5.04 / 320) | (147.4 / 4368) | ( 131.9 / 1051) |  |  |  |
| **actual total flight time [s]** |  | 86.9 | 87.1 | 104.0 | *0.85* | *71.5* | *0.43* |  | 9.64 | 178.4 | 49.2 | *3.99* | *10.0* | *0.053* |
|  |  | (64.9 / 116.4) | (64.5 / 117.4) | (75.6 / 143.2) |  |  |  |  | (2.00 / 46.4) | (49.4 / 643.5) | (22.4 / 107.8) |  |  |  |
| **total gap duration [s]** |  | 36.9 | 59.3 | 35.9 | *0.36* | *77.0* | *0.67* |  | 6.10 | 407.1 | 107.6 | *1.12* | *10.0* | *0.36* |
|  |  | (14.05 / 94.3) | (21.86 / 157.9) | (12.09 / 102.9) |  |  |  |  | (-0.89 / 443) | (12.89 / 11996) | (12.70 / 860) |  |  |  |
| **mean gap duration [s]** |  | 24.1 | 37.5 | 23.9 | *0.39* | *76.3* | *0.68* |  | 6.07 | 177.8 | 54.0 | *0.92* | *10.0* | *0.43* |
|  |  | (9.82 / 57.21) | (14.99 / 91.62) | (8.71 / 62.91) |  |  |  |  | ( -0.81 / 263) | ( 8.29 / 3438) | (8.00 / 335) |  |  |  |
| **number of gaps** |  | 1.12 | 1.34 | 1.24 | *0.23* | *80.0* | *0.79* |  | 0.43 | 2.93 | 1.44 | *1.63* | *7.60* | *0.26* |
|  |  | (0.74 / 1.58) | (0.89 / 1.89) | (0.80 / 1.80) |  |  |  |  | (-0.38 / 2.30) | (0.95 / 6.91) | (0.51 / 2.93) |  |  |  |
| **max. velocity [ms^-1^]** |  | 7.68 | 7.29 | 7.28 | *0.52* | *73.1* | *0.60* |  | 5.16 | 7.17 | 7.90 | *1.15* | *10.0* | *0.36* |
|  |  | (6.75 / 8.74) | (6.42 / 8.27) | (6.35 / 8.34) |  |  |  |  | (3.16/ 8.44) | (4.80 / 10.70) | (6.18 / 10.10) |  |  |  |
| **mean velocity [ms^-1^]** |  | 3.17 | 3.46 | 2.98 | *2.80* | *73.0* | *0.07* |  | 3.03 | 2.24 | 3.09 | *0.84* | *10.0* | *0.46* |
|  |  | (2.75 / 3.60) | (3.03 / 3.90) | (2.53 / 3.44) |  |  |  |  | (1.68 / 4.38) | ( 1.14 / 3.35) | (2.41 / 3.77) |  |  |  |
| **max. acceleration [ms^-2^]** |  | 3.40 | 3.57 | 3.25 | *0.48* | *75.5* | *0.62* |  | 2.11 | 3.55 | 3.57 | *1.33* | *2.00* | *0.51** |
|  |  | (2.94 / 3.93) | (3.07 / 4.15) | (2.76 / 3.82) |  |  |  |  | ( 0.93/ 4.79) | (1.82 / 6.94) | (2.37 / 5.38) |  |  |  |
| **mean acceleration [ms^-2^]** |  | 1.05 | 1.17 | 0.97 | *2.94* | *73.8* | *0.06* |  | 1.05 | 0.76 | 1.05 | *0.74* | *10.0* | *0.50* |
|  |  | (0.91 / 1.19) | (1.02 / 1.32) | (0.82 / 1.13) |  |  |  |  | (0.54/ 1.56) | (0.34 / 1.17) | (0.80 / 1.31) |  |  |  |
| **approachiness** |  | 0.43 | 0.41 | 0.37 | *0.78* | *77.5* | *0.46* |  | 0.44 | 0.16 | -0.15 | *13.9* | *8.10* | ***0.002*** |
|  |  | (0.36 / 0.49) | (0.34 / 0.48) | (0.30 / 0.44) |  |  |  |  | (0.21 / 0.67) | (-0.04 / 0.35) | (-0.29 / -0.02) |  |  |  |
| **straightness** |  | 0.59 | 0.55 | 0.52 | *0.60* | *77.1* | *0.55* |  | - | - | - | - | - | - |
|  |  | (0.50 / 0.69) | (0.46 / 0.65) | (0.43/ 0.62) |  |  |  |  |  |  |  |  |  |  |
