# Supplementary material for: So Near and Yet So Far: Harmonic Radar Reveals Reduced Homing Ability of Nosema Infected Honeybees
Source: PLoS One. 2014 Aug 6;9(8):e103989. doi: 10.1371/journal.pone.0103989 (PMC4123971; doi:10.1371/journal.pone.0103989)
Supplement: File S1 — Supporting information. Detailed description of the experimental prodedures, data analyses and additional statistical analyses of the results. (DOCX) [file pone.0103989.s003.docx]

**Supplementary material**

**Detailed Materials and Methods**

***Bee quarantine***

All experimental bees originated from three donor colonies (DCs) from the Isle of Colonsay, UK (56° 3’ 56’’ N, 6° 12’ 24’’ W), which is currently devoid of *Varroa destructor* so that potentially confounding effects of this mite or its associated viruses were minimized. These colonies were quarantined over the entire period of the experiment in a large flight cage (3m x 4m x 9m), in which foragers were allowed to freely forage on 240 flowering *Phacelia tanacetifolia* plants, five feeders with pollen (UV-radiated for 24 h to de-contaminate from pathogens) and sucrose solution (40% weight/weight or w/w).

***Preparation of Nosema-inoculum***

Initial *N. ceranae* spores were obtained from the mid-guts of infected bees of a free foraging *A. mellifera* colony located in Halle/S., Germany, in July 2011. As *N. ceranae* spores are cold sensitive and therefore cannot be freeze-stored, spores had to be continuously propagated in live bees. We used approximately 30 - 50 adult bees (stock-bees) from a quarantined *Nosema*-clean colony, which were collected every 3 - 4 weeks. These were inoculated with a 40% sucrose solution containing approximately 150,000 spores/bee administered through a bulk-feeder, and bees were subsequently kept in holding cages in an incubator.

The *Nosema*-inoculum was then obtained from five to seven freshly dissected guts of the above stock-bees. Guts were manually ground up and dissolved in 1ml isotonic saline solution (0.9% w/w). The gut extract was filtered through cotton wool to remove large debris. The resulting filtrate was subsequently purified by triangulation [1] as follows. After an initial centrifugation of the filtrate (5000 rcf for 5 min), the supernatant was discarded, the pellet re-suspended in 1ml saline (0.9%) and centrifuged at 30 rcf for 3 min. The resulting supernatant was transferred to an empty 1.5 ml Eppendorf tube, while the remaining pellet was again re-suspended in 1 ml saline. Both tubes were subsequently centrifuged at 30 rcf for 3 min. This procedure was repeated eight times, resulting in nine tubes of gut extract of various compositions. In a final centrifugation step of all nine tubes (at 5000 rcf for 5 min), all particles were sedimented and the supernatant was discarded. After triangulation, the pellets from tubes three to five consisted of > 95% spores [1]. These pellets were suspended in saline solution and merged into a total of 1 ml purified spore solution. Spore densities were determined using a haemocytometer (Neubauer, magnification 400 x).

After purification and quantification, the spore solution was mixed with a 40% (w/w) sucrose solution, forming the treatment inoculum (hereafter Nc). Then 100,000 *N. ceranae* spores (ca. 10 µl solution) were fed using a pipette to a bee harnessed in a cage. The spore samples were repeatedly checked for species composition using a species-specific PCR [2].

***Data Analysis***

Based on the recorded radar signals, tracks were manually digitalized using a custom-made TAS - Track Analysis Software V1.0 (by Shane Hatty, ©Rothamsted Research 2008). Positional information was transformed from polar coordinates (range, angle) to Cartesian coordinates (x, y) for further analysis.

The start of a flight track was defined by the release time (recorded during experimental procedures) and the coordinates recorded shortly before release (see above). Successful homing tracks ended with the bee being observed or caught at the hive entrance, with the last position being identical to the hive position. Tracks of non-returning bees were defined in terms of their last recorded position after which no signal was recorded for a further 45 min (Fig 2).

Though ideally the bee’s position would be recorded every three seconds, tracking information can be discontinuous (i.e. causing larger read intervals) due to either an obscured flight path or the bee stopping *en route*. For the former, the coordinates flanking the obscured part of the flight were connected by a straight line, resulting in a conservative underestimate of the actual covered flight distance. In contrast, a genuine discontinuation of the flight (hereafter referred to as “stop”) was assumed when both the velocity was below 0.33 m/s and the total distance covered during the putative stop time was below 10 m (i.e. twice the resolution of the radar, accounting for the bee flying below radar-detection on approach to and take off from a resting position).

From the tracks we extracted two types of parameter using Matlab V7 ([www.mathworks.com](http://www.mathworks.com)):

1. parameters related to the actual flight characteristics of the bee irrespective of its homing success (hereafter Flight parameter):

mean and maximum flight speed (ms^-1^),

mean and maximum acceleration (ms^-2^),

track straightness with reference to the last track coordinate (shortest possible track length/actual track length; see [3]),

mean and maximum turning angle (degree),

proportion of flight time of total track duration,

number of stops,

total and mean stop duration (s)

1. parameters characterizing the spatial and temporal dimensions of the track itself (hereafter Track parameter):

track length (m),

track duration (s),

total flight time (s),

total and mean stop duration (s)

directionality towards the hive (A, see below)

As track parameters, in contrast to flight parameters, are affected by homing success, we only compared track parameters between treatment groups for bees that successfully returned to the colony. Total and mean stop duration (s) could not be clearly allocated to either of the groups and were included in the analysis of both types of parameters.

The navigational abilities of the tracked bees were assessed through the goal-directedness of the homing flights towards the hive (hereafter referred to as “Approachiness” A) based on the angle (α) formed between a bee’s position (t_n_), the position of the hive and the next recorded bee position (t_n+1_), which is 0° for a direct approach, 180° for flights directly away from the hive and maximal for equidistant orbiting flights based on the laws of trigonometry. The resulting A – values range from A = 1 for a direct approach to A = -1 for a straight flight away from the hive, with A = 0 representing an equidistant orbiting flight. A – Values were calculated for every two consecutive positional records of a bee within a track using Matlab V7 (www.mathworks.com). Individual A-values were weighted for the proportion of the total track covered between the two positions and averaged for the total track.

Additionally, we analysed the heading direction (as measured from the release site, Fig 3 and 4) when the bee flew beyond 60m from the release site for the first time (half of the homing distance) or at the last known position, in cases where the tracks did not progress beyond 60m. This truncation allowed a comparison of the spatial distribution of returning and non-returning bees, including initial orientation loops. Within the 60m radius, the proportional distribution of flight distances and flight times among the four quadrants of the coordinate system were assessed.

We also tested the utilization of available landmarks by the bees as a proximate measure of orientation ability. We compared the actual flight paths to two hypothetical homing flight scenarios: S1) the direct straight flight from the release site (RS) to the hive, and S2) a flight along the available landmarks following the field margin connecting release site to hive (Fig. 1b). For both hypothetical paths, we calculated the coordinates of 100 equidistantly spaced markers representing the path. Using the coordinates of the bee’s position during the homing flight, we determined the distance to the nearest possible marker under either scenario. Both shortest total and mean distances of a bee’s flight under either scenario were used to assign the realized flight to either hypothetical flight path.

**Supplementary Results**

***Effects of Infection Intensity***

Though there was no significant correlation between spore load and the measured flight parameters, we found a positive, yet non-significant, trend between normalized spore load and both mean and total stop duration. Both regressions were not significant for the full dataset (spore density vs. mean stop duration / total stop duration: *d.f*. _regression, total_ = 1, 20 / 1, 20; *s.s.* = 1.661 / 2.28; *v.r.* = 3.55 / 3.33; *p* = 0.075 / 0.084) and accounted for 11.3% and 10.4% of the total variation in mean and total stop duration, respectively. However, in both cases two data points returned a high Cook-statistics, indicating a disproportionately strong effect on the overall regression.

After at least one of two data-points had been excluded, spore load was significantly positively correlated with stop duration (mean / total stop duration with both outliers excluded: *d.f.* _regression, total_ = 1, 18 / 1,18; *s.s.* = 6.76 / 4.02; *v.r.* = 14.21 / 11.14; *p* = 0.002 / 0.004). Here the variation that could be explained by spore load increased to 36% and 42.3% (Fig. S1).

***Flight and Track Characteristics***

Flight characteristics varied widely within treatment groups (TR). Accounting for homing success (TR x HS) in the statistical analyses none of the parameters differed significantly between the *Nosema* infected bees and the control bees (Table 1).

Also, comparing the treatment groups for returning and non-returning bees separately (Table S1) we did not find any significant differences. This indicates that an infection with *N. ceranae* does not affect the characteristics of the flight itself. Analysing all flight parameters together in a multivariate approach (Canonical Variate Analysis (CVA)) confirmed that flight parameters do not allow a clear separation of the treatment groups (Fig. 6a).

For the track parameters, we found a similar pattern for the global and the separate analyses. In both tests Approachiness (a measure of directionality of hive approach) was the only parameter that showed a highly significant reduction from 0.30 and 0.42, for C_GE_ and C_S_ bees respectively, to 0.11 in the Nc-group (*F^TR^_2, 86.3_* = 9.98, *p* < 0.001) (Tables 1 and S1). This effect is expected as the overall A-value increases towards the end of a successful homing flight and thus we expect reduced values for non-returning bees, which were heavily represented in the Nc-group. In comparison with all other parameters, only “Approachiness” and actual total flight time showed some separation of the Nc-group from the controls (Fig. 6b). Both of these parameters are greatly affected by homing success.

**References**

1. Cole R (1970) The application of the “triangulation” method to the purification of *Nosema* spores from insect tissues. Journal of Invertebrate Pathology 15: 193-195.

2. Fries I, Chauzat M-P, Chen Y-P, Doublet V, Genersch E, et al. (2013) Standard methods for *Nosema* research. Journal of Apicultural Research 52: 1-28.

3. Osborne JL, Clark SJ, Morris RJ, Williams IH, Riley JR, et al. (1999) A landscape-scale study of bumblebee foraging range and constancy, using harmonic radar. Journal of Applied Ecology 36: 519-533.
